# Toward automatic C-arm positioning for standard projections in orthopedic surgery

**DOI:** 10.1007/s11548-020-02204-0

**Published:** 2020-06-12

**Authors:** Lisa Kausch, Sarina Thomas, Holger Kunze, Maxim Privalov, Sven Vetter, Jochen Franke, Andreas H. Mahnken, Lena Maier-Hein, Klaus Maier-Hein

**Affiliations:** 1 Division of Medical Image Computing, German Cancer Research Center, Heidelberg, Germany; 2 Division of Computer Assisted Medical Interventions, German Cancer Research Center, Heidelberg, Germany; 3 Imaging and Therapy Systems Division, Siemens Healthineers, Erlangen, Germany; 4 Medical Imaging and Navigation in Trauma and Orthopedic Suregery Research group, BG Trauma Center, Ludwigshafen, Germany; 5 Division of Diagnostic and Interventional Radiology, Universitätsklinikum Marburg, Marburg, Germany

**Keywords:** Pose estimation, Fluoroscopic imaging, C-arm positioning, Standard projection

## Abstract

**Purpose:**

Guidance and quality control in orthopedic surgery increasingly rely on intra-operative fluoroscopy using a mobile C-arm. The accurate acquisition of standardized and anatomy-specific projections is essential in this process. The corresponding iterative positioning of the C-arm is error prone and involves repeated manual acquisitions or even continuous fluoroscopy. To reduce time and radiation exposure for patients and clinical staff and to avoid errors in fracture reduction or implant placement, we aim at guiding—and in the long-run automating—this procedure.

**Methods:**

In contrast to the state of the art, we tackle this inherently ill-posed problem without requiring patient-individual prior information like preoperative computed tomography (CT) scans, without the need of registration and without requiring additional technical equipment besides the projection images themselves. We propose learning the necessary anatomical hints for efficient C-arm positioning from *in silico* simulations, leveraging masses of 3D CTs. Specifically, we propose a convolutional neural network regression model that predicts 5 degrees of freedom pose updates directly from a first X-ray image. The method is generalizable to different anatomical regions and standard projections.

**Results:**

Quantitative and qualitative validation was performed for two clinical applications involving two highly dissimilar anatomies, namely the lumbar spine and the proximal femur. Starting from one initial projection, the mean absolute pose error to the desired standard pose is iteratively reduced across different anatomy-specific standard projections. Acquisitions of both hip joints on 4 cadavers allowed for an evaluation on clinical data, demonstrating that the approach generalizes without retraining.

**Conclusion:**

Overall, the results suggest the feasibility of an efficient deep learning-based automated positioning procedure, which is trained on simulations. Our proposed 2-stage approach for C-arm positioning significantly improves accuracy on synthetic images. In addition, we demonstrated that learning based on simulations translates to acceptable performance on real X-rays.

## Introduction

Mobile fluoroscopic imaging is used to guide interventions in orthopedic and trauma surgery and to evaluate the success of the fracture reduction and implant placement. An essential task in image-guided surgery is the generation of a correct standard projection of the anatomy for medical verification [1]. The correct projection corresponds to a specific pose of the C-arm relative to the patient’s positioning. It is challenging to obtain the desired view due to variabilities in patient placement and because the internal anatomy is not visible from outside. Incorrect projections can result in overlays of anatomical structures and ambiguities due to the effects of projective simplification, thus increasing the risk of overlooked errors. Examples of critical errors include the malunion of fractures, leading to functional impairment and in the worst case, requiring a subsequent intervention at increased rates of complication.

In current practice, repeated or continuous fluoroscopy images are acquired for C-arm positioning, with the radiological technician following a trial-and-error approach. Time consumption and radiation exposure for patients and personnel are largely dependent on the experience of the operator. In addition, due to lacking standards and common definitions, the resulting standard projections are highly surgeon dependent. Rikli et al. discovered in a retrospective assessment study that only $78.8\%$ of the lateral post-implant projection images were classified as correct lateral standard projection, resulting in not assessable fracture reduction ($10.3\%$) or not assessable implant position ($7.4\%$) [19].

In general, state of the art assistance systems either require external tracking hardware, preoperative CT scans or manual landmark selection, which so far limited broad clinical applicability.

**Fig. 1:**
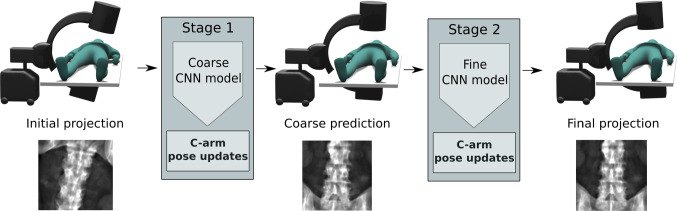
Concept overview: For efficient C-arm positioning toward anatomy-specific standard projections, we propose a 2-stage deep learning-based regression approach. In subsequent stages, the 5 DoF relative pose update to reposition the C-arm toward the desired standard projection is predicted, directly from an initial 2D projection

In this work, we aim at tackling the problem of C-arm positioning for standard projections without additional technical burden, directly working on the 2D projection images just as the operator. This task is severely under-constrained in theory, as multiple shape configurations can produce the same image projections. We propose learning the necessary anatomical hints for efficient C-arm positioning from *in silico* simulations, leveraging masses of 3D CTs. Specifically, as illustrated in Fig. 1, we propose a CNN regression model that predicts, directly from 2D projections, the 5 degrees of freedom (DoF) system parameter updates toward the desired standard projection, thus assisting the current manual procedure. Training on simulated images uniquely provides ground truth pose labels, which are not available in real fluoroscopic data. In our analysis, we focused on two very dissimilar anatomies: the proximal femur and the spine (fourth vertebra). We considered two standard projections for each anatomical region, demonstrating the potential applicability of the approach to various scenarios. Finally, we demonstrate that the approach also generalizes to unseen cadaver X-rays without retraining.

## Related work

Fluoroscopy simulation methods for pose guidance presented in the literature rely on a preoperative CT scan or an anatomical atlas. In addition, they require an external tracking system to estimate the pose of the patient relative to the C-arm [4, 9]. The integration of tracking systems in the clinical workflow is challenging, mainly due to the additional hardware setup and the related line-of-sight requirements. Consequently, the application of these approaches is currently focused on surgical training purposes. Recently, a 3D–2D registration between a preoperative CT scan and initially acquired radiographs was shown to allow the generation of virtual fluoroscopy images in real time [6, 7]. A preoperative scan is still required, though, and all the mentioned approaches are designed to support the surgeon, but not to directly deliver the optimal pose. Rodas et al. presented a Monte Carlo approach to optimize the pose of a C-arm in a defined neighborhood around the known standard direction while reducing the radiation exposure [20]. This so-called standard direction, however, depends on the positioning of the patient relative to the C-arm and on the patient-specific anatomy, which is unknown in general. Recently, Haiderbhai et al. presented a user interface for automatic C-arm positioning [10]. The surgeon can define an optimal view based on simulated X-rays from a preoperative CT. This is converted to a C-arm position using inverse kinematics [16, 22]. It requires a registration of the preoperative CT and the patient each time either is moved. Maier et al. showed the potential of using inertial measurement units for object motion correction in C-arm imaging [15].

Landmark-based registration is an alternative approach to image-guided positioning of C-arms [3]. It comes with the limitation of requiring two distinct projection images and visible corresponding landmarks, manually depicted by the surgeon during the intervention.

Deep learning-based C-arm localization methods, formulated as 2D-3D registration task, which requires an available 3D volume, were presented by Miao et al. [17]. They formulate the task as a Markov decision process and propose a multi-agent system to handle various artifacts in 2D X-ray images. Pose regression without a preoperative scan has been proposed for slice transformation prediction with respect to a canonical atlas coordinate frame by Hou et al. [11]. On digitally reconstructed radiographs (DRR) computed from preprocessed CTs (identical intensity ranges, spacing, translation), they achieved sufficiently accurate performance (average translation error of 106 mm and $5.6\,{}^{\circ }$ plane rotation for healthy patients) for the task of robust initialization of X-ray to CT registration. On the contrary, in our application, we have to deal with the challenges of varying image contrast, due to patient anatomy or different acquisition doses and require higher accuracy in translation prediction to assure the region of interest is centered in the FoV. The closest related work is machine learning-based pose estimation for mobile X-ray imaging by Bui et al. [5]. They focus on 6 degrees of freedom modeling plus industrial applications where the existence of a precise 3D CAD model can be assumed as prior knowledge, serving as additional reprojection constraint. The experiments show that neural networks outperform other regression approaches in the prediction of object poses from simulated X-ray projections, with high pose prediction accuracy for object-specific models and decreasing accuracy for more generic CNN models trained on sets of different objects.

The object-specific case is closely related to our approach, with the difference being the inter-subject anatomical variation that we have to deal with additionally.

The remaining part of the paper is organized as follows: “Materials and methods” section introduces the methodological details of our proposed approach. Experimental results on *in silico* and real projections are presented and discussed in “Experimental results” section.

## Materials and methods

For an efficient positioning of the C-arm toward an anatomy-specific standard projection, we focus our analysis exemplarily on two different anatomical regions, the proximal femur (PF) and the fourth lumbar vertebra (LV4). With an appropriate amount of available training data and well-defined standard projections, the proposed method in principle can be directly applied to any other anatomical region. A proper evaluation of reduction and implant position and thus the success of surgical intervention requires acquisition of anatomy-specific standard projection. We focused our analysis on the *AP* projection (X-rays traveling from anterior to posterior) and the *Lateral* projection (X-rays from left to right side of the body or vice versa) since these are the most frequently acquired and the most relevant for hip and spine procedures (cf. Fig. 7, last row).

This section presents the mobile C-arm device and its DoFs (“Mobile C-arm device” section), the framework for generating a ground truth reference and in silico training data (“Generation of training data” section), and the proposed approach for automatic C-arm positioning toward a specific standard projection (“Pose regression framework” section).

### Mobile C-arm device

**Fig. 2:**
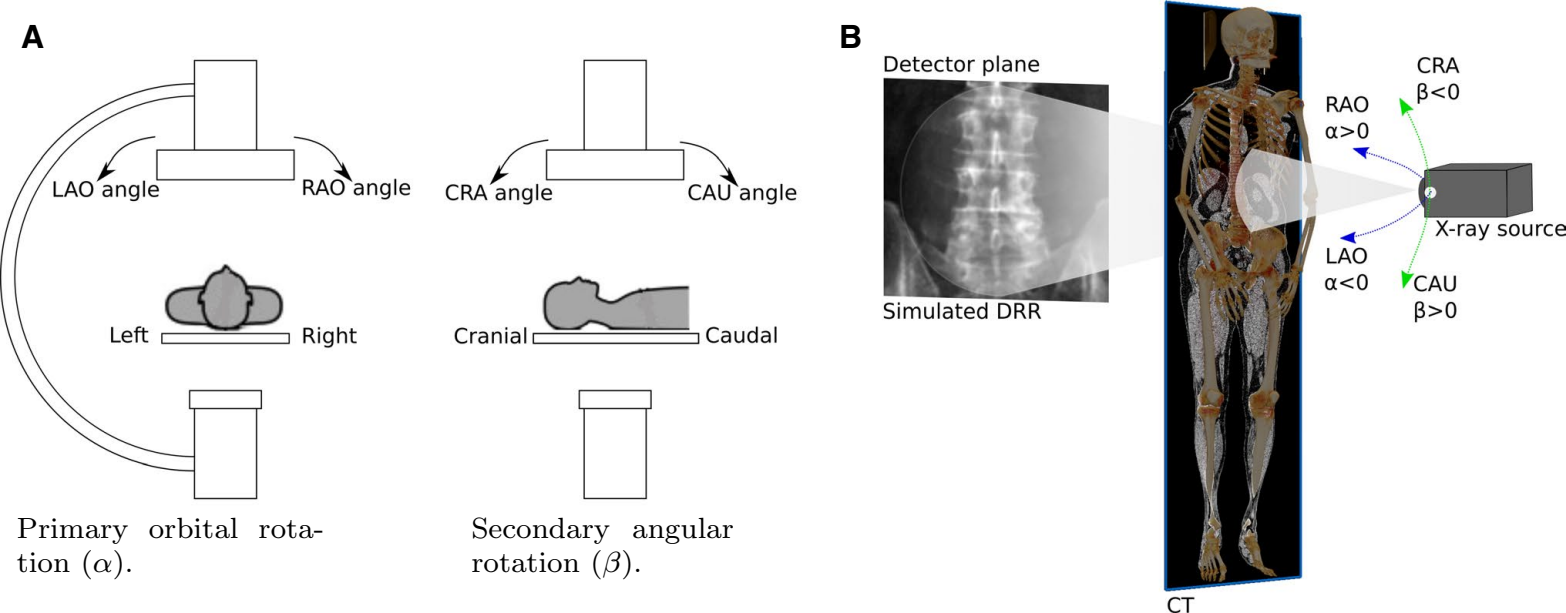
Rotational DoF of the C-arm (**a**) and simulation of training data (**b**)

The mobile C-arm is a C-shaped imaging system with an X-ray source and a flat panel detector at its ends. The C-arm has six DoF, three translational and three rotational. The translational parameters influence the image center and scale of the projection image. The rotational DoF is depicted in Fig. 2a: Firstly, the rotations within the plane of C-arm gantry are referred to as orbital rotation and denoted by $\alpha$ (LAO). Secondly, rotations perpendicular to the plane of the C-arm gantry are referred to as angular rotations and denoted by $\beta$ (CRA). Thirdly, the image rotation in the detector plane is denoted by $\gamma$. We consider the translation parallel to the detector plane and omit the translation along the beam direction, which only influences the scale of the projection, assuming that the structure of interest is initially located approximately midway between source and detector. The C-arm setup allows for acquiring images of a patient from any projection angle only limited by the patient anatomy.

### Generation of training data

Due to the unavailability of annotated X-ray images, training data is synthetically generated from full-body CTs acquired at different institutions and scanners. Initially, the CTs were cropped to a region of interest (ROI) around the proximal femur and the fourth lumbar vertebra, respectively. This prevents other extremities from overlaying the projection image, which is also ensured in real C-arm acquisitions by the patient placement.

*Definition of ground truth reference* Two independent clinical experts defined the two reference standard projection planes for each of the anatomical regions. To increase the amount of training data, for the proximal femur, both lateralities were included, one laterality was mirrored before training data generation. Furthermore, for the *AP* standard only CT volumes with a slice thickness and distance below 3 mm were considered. This results in 81 cases for *AP* and 109 cases for *Lateral*. For the lumbar spine, 47 patients were considered for both standards. An interactive tool for simulating the DRR preview was implemented in MITK using interactive plane translation and rotation [18].

*Simulation framework* For generation of training data, the source and detector pose were varied around the reference C-arm pose (Fig. 2b), defined by the standard planes. For a realistic forward projection of the 3D CT anatomy, we use DeepDRR [21], which computes energy- and material-dependent attenuation images that are converted to synthetic X-rays. The choice of DeepDRR simulation is motivated by its generalization capabilities toward clinically acquired X-rays as shown by [2, 8]. We defined the system parameters according to a Siemens Cios $\hbox {Spin}^{\textregistered }$ robotized C-arm. It has a $300\times 300\,mm^2$ detector at $1952\times 1952$ pixel resolution and a source–detector distance of 1164 mm. The DRRs are simulated for a reduced resolution of $512\times 512$ pixels to allow faster computation and are labeled according to their pose distance to the standard beam direction.

**Fig. 3:**
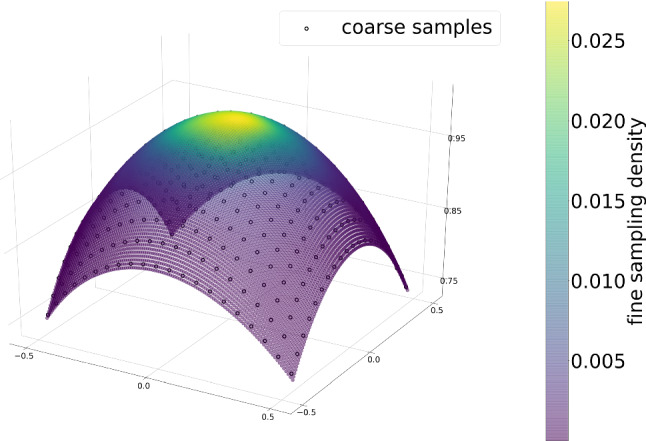
Coarse and fine angular sampling strategies for data generation around the standard beam direction

**Fig. 4:**
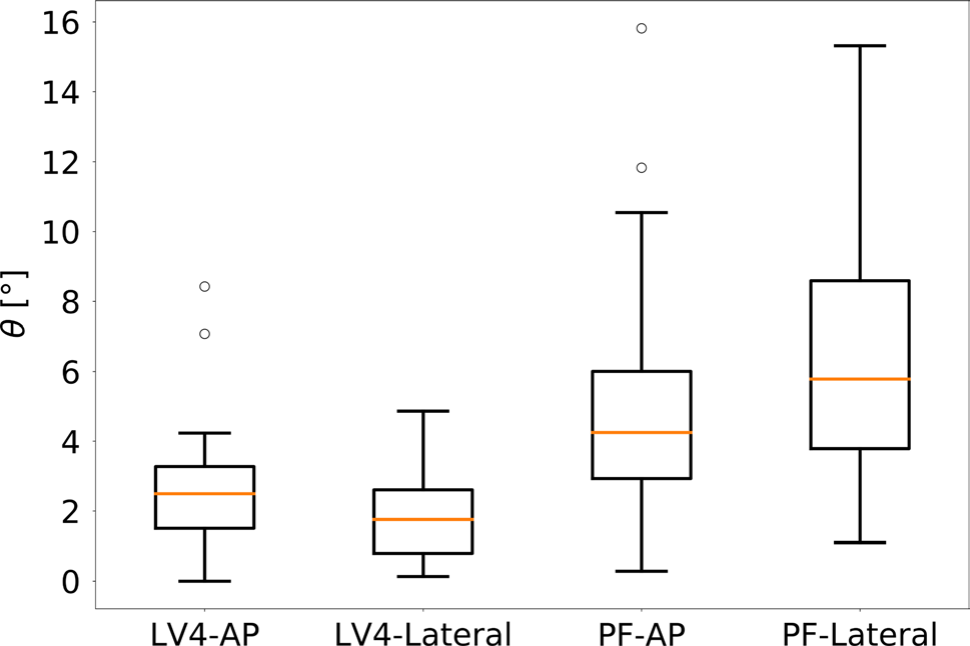
Inter-rater variance of standard beam direction across two independent expert definitions for different patients under the additional prior of pairwise plane orthogonality

*Sampling strategies* We sampled projections on two spherical segments for training the two networks (Figs. 1, 3): For coarse positioning, we cover a broader range with $\alpha ,~\beta \in [-30,30]\,{}^{\circ }$ in steps of $3\,{}^{\circ }$ centered around the standard beam direction. The training set of uniformly sampled projections in $\alpha -\beta$ plane is denoted by $\mathcal {X}=\left\{ \mathbf {x}_i\right\} _i$ with projections $\mathbf {x}_i \in \mathbb {R}^{512 \times 512}$. The training set for subsequent fine positioning was simulated with rotational parameters sampled from a Gaussian distribution so that the coarse angular region is covered with 99 % confidence and is denoted by $\mathcal {X}^{'}$.

*Augmentation* The in-plane rotation $\gamma$ was fixed during the simulation and was augmented online during training on the simulated dataset, thereby limiting the number of simulations and hence computation time. For the coarse set $\mathcal {X}$, image rotation was augmented with rotation angles randomly sampled from a uniform distribution $\gamma \in \mathcal {U}(-180,~180)$ and for the fine set $\mathcal {X}^{'}$, from a Gaussian distribution $\gamma \in \mathcal {N}(\mu =0,\sigma ^2=\left( 30/2.576\right) ^2)$, respectively. The image rotation is followed by a center crop to eliminate border interpolation effects due to image rotation. We assume that detector rotation can be approximated by image rotation at reduced image resolution. Non-isotropic radiation patterns due to collimation are neglected by this approximation. In practice, pure gantry rotation along this axis requires a C-arm base movement. We decided to model this rotation in addition to the orbital and angular C-arm rotations to gain independence to variable initial C-arm to patient orientations, depending on the angle in which the C-arm is moved toward the patient table. Translation in the detector plane was simulated by augmenting random crop transforms with translation of the image center $t\in [-50,50]^2\,mm^2$. Thus, the labeled training set is given by $\mathcal {S} = \{(\mathbf {x}_1,\mathbf {p}_1),\ldots ,(\mathbf {x}_N,\mathbf {p}_N)\}$ with $\mathbf {p}_i=[\alpha _i,~\beta _i,~\gamma _i,~t_x,~t_y] \in \mathbb {R}^5$, with projections $\mathbf {x}_i$ rescaled to a final resolution of $256\times 256$ pixel. Additional online augmentations were applied during training to increase the variety of training data: We used image scaling $s \in [0.8, 1]$ which approximates variation of the source-to-isocenter distance or bone sizes. We excluded larger scale coefficients resembling moving the source closer to the anatomy, because this results in a smaller field-of-view and a higher patient dose, which is not clinical practice. Moreover, contrast augmentation was performed by modifying each pixel *p* according to $p = (p-\mu )*c + \mu$ for image-wide $c \in [0.75, 1.25]$, where $\mu$ denotes the mean of the image [13].

### Pose regression framework

**Fig. 5:**
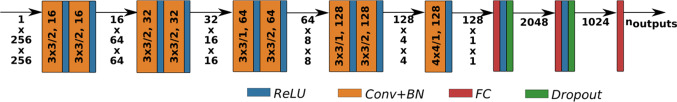
CNN architecture $f(\cdot ,\,\cdot )$: Four convolutional blocks followed by three fully connected layers, inspired by [5]

For positioning the C-arm relative to a specific standard projection, we propose a 2-stage sequential model based on CNN regression (Fig. 1), estimating the pose update $\hat{\mathbf {p}} = [\hat{\alpha },~\hat{\beta },~\hat{\gamma },~\hat{t_x},~\hat{t_y}]$ for a given initial projection. Both stages use the same network architecture shown in Fig. 5, which is a modified version of PoseNet [5] with a larger input size, batch normalization after each convolutional layer and additional ReLU activation after each 2nd convolutional layer, which showed to improve the convergence during training. The networks are trained separately on different sampled training sets $\mathcal {X}$ for coarse and $\mathcal {X}^{'}$ for fine positioning, respectively. Before training, the input data is range normalized to the [0, 1] interval and preprocessed by a negative Log transform that converts the data from intensity to line integral domain, decreasing the dynamic range. The output is again range normalized to the $[-1, 1]$ interval. While for the out-of-plane rotations the radial Euler angles are predicted directly, for the coarse in-plane rotation the target rotation is converted to sin/cos-space and two values are regressed, which results in a continuous Loss function, which can handle the circularity. Thus, the coarse network has one additional output neuron and is optimized employing a weighted L2 Loss function given by

$$\begin{aligned}&\mathcal {L}_{coarse}(\mathbf {x}_i) \\&\quad = \mathcal {L}_{out-of-plane}(\mathbf {x}_i) +\mathcal {L}_{in-plane}(\mathbf {x}_i) + w \mathcal {L}_{detector-t}(\mathbf {x}_i)\\&\quad = \Vert \underbrace{\hat{\alpha }-\alpha }_{:=d\alpha } \Vert ^2 + \Vert \underbrace{\hat{\beta }-\beta }_{:=d\beta } \Vert ^2 + \Vert \hat{c}_\gamma -\cos {\gamma } \Vert ^2 + \Vert \hat{s}_\gamma -\sin {\gamma } \Vert ^2 \\&\qquad + w^2 (\Vert \underbrace{\hat{t_x} - t_x}_{:=dt_x} \Vert ^2 + \Vert \underbrace{\hat{t_y} - t_y}_{:=dt_y} \Vert ^2) , \end{aligned}$$

where $w=\pi \cdot (180)^{-1}$ is a weighting factor to equally penalize orientation (${}^{\circ }$) and translation (mm) error and $\hat{\alpha },~\hat{\beta },~\hat{c}_\gamma ,~\hat{s}_\gamma ,~\hat{t_x},~\hat{t_y}$ are outputs of the coarse network. The predicted in-plane rotation can be recovered by $\hat{\gamma }={{\,\mathrm{atan2}\,}}(\hat{s}_\gamma \cdot ( \hat{s}_\gamma ^2+\hat{c}_\gamma ^2) ^{-\frac{1}{2}},~\hat{c}_\gamma \cdot ( \hat{s}_\gamma ^2+\hat{c}_\gamma ^2) ^{-\frac{1}{2}} )$. The fine positioning network is trained accordingly with a weighted L1 Loss, which penalizes pose errors linearly. One individual network is trained for each of the anatomy-specific standard projections.

The models were implemented using PyTorch and trained with a 6GB GeForce GTX 1060 graphics card. The networks were optimized using the Adam optimizer [12] with a learning rate of $\eta =10^{-4}$ and batch size 64 for 1500 iterations each with 50 batches. The average time of pose prediction for a single image was around 3.8 ms.

### Real X-rays

For evaluation of our proposed method, we performed a study on real X-rays of both hips of 4 cadavers. This dataset was acquired with a Siemens Cios $\hbox {Spin}^{\textregistered }$ mobile C-arm system during surgical courses for physicians and was available retrospectively. It serves as test dataset for the first stage of the proximal femur application which can be evaluated offline. The datasets consist of manually acquired standard projections (*AP*, *Lateral*), an X-ray projection sequence and a corresponding reconstructed 3D volume of each hip. One dataset was excluded due to a metal implant.

## Experimental results

The proposed pose regression framework was evaluated on datasets that were previously not seen by the model, thus validating the ability to handle intra-class anatomical variation. For accuracy evaluation, we report the angle between the principal rays $\theta$ of the ground truth standard beam direction $\nu :=[\sin \alpha , -\cos \alpha \cdot \sin \beta , \cos \alpha \cdot \cos \beta ]^T$ and the predicted beam direction $\hat{\nu }:=[\sin \hat{\alpha }, -\cos \hat{\alpha }\cdot \sin \hat{\beta }, \cos \hat{\alpha }\cdot \cos \hat{\beta }]^T$, given by $d\theta :=\arccos {(\cos \nu \cdot \cos \hat{\nu })}$, the absolute error of rotation $d\alpha$, $d\beta$, $d\gamma :=\min \left\{ |\hat{\gamma }-\gamma |,360-|\hat{\gamma }-\gamma |\right\}$ and the Euclidean distance of image translations $dc:=\Vert [dt_x, dt_y]^T\Vert$.

This section evaluates the precision of our underlying ground truth (“Precision of reference standard projections” section) and presents experiments for C-arm positioning conducted on synthetic (“Pose estimation for standard projections on synthetic x-rays” section) and real X-rays (“Pose estimation for standard projections on real x-rays” section).

### Precision of reference standard projections

To measure the quality of our reference data, we first evaluated the inter-rater variance of the two clinical experts, who independently defined the standard planes in 3D CT volumes. Inter-rater variations were particularly high for the PF-AP standard, where multiple beam directions can result in similar projections. Optimally, the standard projections are assumed to be pairwise orthogonal. Hence, to assure optimal ground truth standards, the surgeons defined the two standard directions simultaneously under consideration of pre-defined common guidelines and of the additional prior of pairwise plane orthogonality, resulting in an average inter-rater variance of up to $6.3^{\circ }$ in beam direction (Fig. 4) and up to 9.3 mm average isocenter translation for all considered standard projections (Fig. 5). It is important to state that the derived inter-rater variances are by no means representative for the precision achieved in real clinical scenarios, where the variance is much higher (cp.“Pose estimation for standard projections on real x-rays” section).

**Fig. 6:**
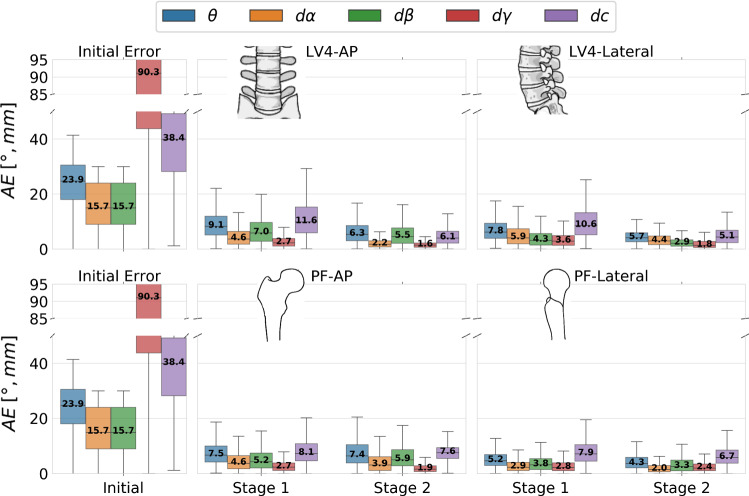
Quantitative evaluation of the sequential pose regression accuracy. The angular error distribution of the principal rays $\theta$ is shown together with the angular error distribution $d\alpha ,\,d\beta ,\,d\gamma$ and the Euclidean distance of translation in the detector plane *dc*

**Fig. 7:**
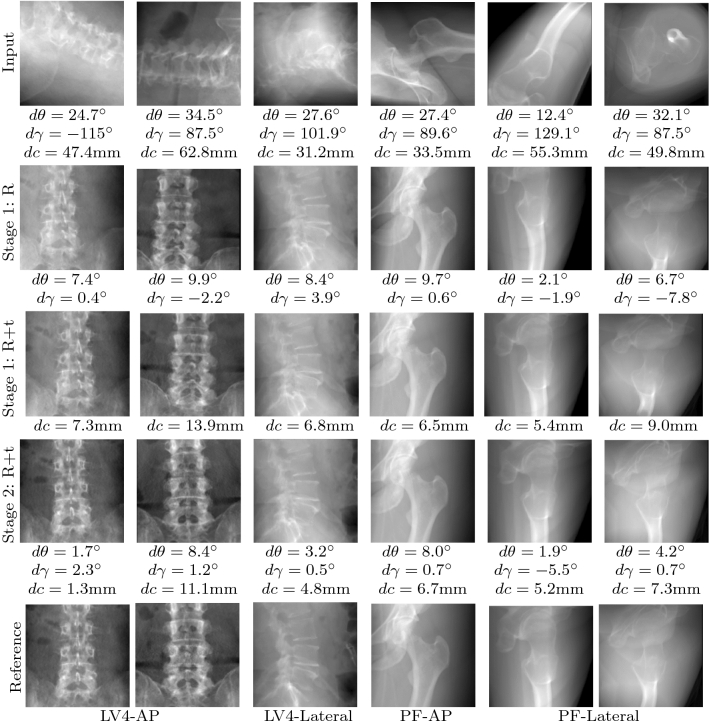
Qualitative evaluation of the sequential pose prediction on synthetic X-rays. For comparison, the last row shows the reference standard defined by the expert

### Pose estimation for standard projections on synthetic X-rays

The main objective of this paper is to demonstrate the feasibility of predicting relative pose updates for a C-arm given one initial projection of a yet unseen patient. The proposed sequential approach (cp. Fig. 1), where the C-arm is iteratively guided toward the desired standard pose, was evaluated on simulated data. The CT data used for simulation was randomly divided into $60\%$ training, $20\%$ validation and $20\%$ test. Figure 6 shows quantitative results on datasets previously not seen by the model: The angular and translational error distribution of the initial projections is shown on the left. Next to that, the distribution of the pose error across the 2 stages separately for all considered standard projections is depicted. Starting from one initial projection, the mean absolute pose error to the desired standard pose is iteratively reduced across different anatomy-specific standard projections. The 2-stage sequential approach with coarse and fine positioning outperforms a baseline approach with a single prediction step. Also, our 2-stage approach showed superior performance compared to iteratively applying one prediction network, confirming the hypothesis that the subdivision in coarse and fine positioning is crucial for the iterative accuracy gain. While the coarse network complies with the task of rough initialization toward the desired standard, the fine network is specialized in small pose adjustments. Our method was able to iteratively improve the projection results toward the desired standard projection as shown visually in Fig. 7.

### Pose variance in clinical practice

In a first step, we analyzed the performance of the surgeon in positioning the C-arm, and thus in acquiring the standard projections manually. To this end, we computed the pose variance of the manually acquired standard projections to a reference plane retrospectively defined in the corresponding 3D scan.

The pose variance in clinical practice was evaluated for the *AP* and *Lateral* projection of the proximal femur, based on the real X-ray data, described in “Real x-rays” section. A surgeon defined the reference standard planes in the 3D scans, similar to the reference CT datasets. The variance between the defined reference standard and the acquired manual standard serves as a measure for precision achieved in real clinical scenarios.

**Fig. 8:**
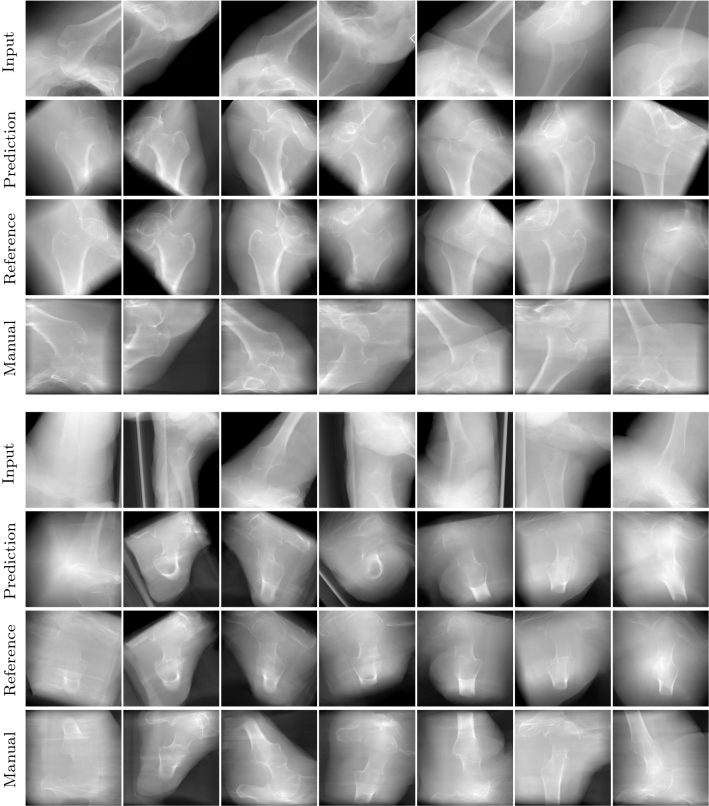
Qualitative evaluation of the pose regressor on real X-rays (first block: *AP* standard, second block: *Lateral* standard). For comparison, the last two rows show the reference standard defined by the expert in the C-arm volume and the manual standard acquired with the C-arm (image rotation implicitly defined by detector orientation)

As a result, we determined an average angle between the principal rays of $25.3 \pm 9.3^{\circ }$ (*AP*) and $15.3 \pm 9.2^{\circ }$ (*Lateral*) and $22.2 \pm 12.6$ mm isocenter offset. It is important to state that the manual and reference standard projections in spite of the orientation variance look visually very similar (Fig. 8, last two rows of each block) and both projections are generally accepted as standards by clinical experts. The increased variance compared to the reference standard is mainly due to two factors: Firstly, while the standard projections in clinical practice are acquired independently, the reference standards were defined using coupled planes to assure lower inter-rater variance and thus higher quality of the reference. Secondly, in clinical practice of proximal femur applications, the surgeons only vary the orbital rotation for the acquisition of proximal femur standard planes. The angular rotation is not altered, but is implied by the initial positioning of the C-arm relative to the patient which is not standardized and not guaranteed to be optimal.

### Pose estimation for standard projections on real X-rays

The pose regression framework for standard projections was qualitatively evaluated on datasets containing real X-rays, described in “Real x-rays” section. For evaluation of our proposed pose estimation for C-arm positioning, we chose a random initial projection from the raw orbital projection series of the C-arm. Thereby, it was assured that the initial projection lies inside the capture range of the CNN regressor. After applying image scaling, mirroring (dealing with the different lateralities) and preprocessing according to  “Pose regression framework” section, the resultant image was fed into the coarse pose regressor. The X-ray simulator can be positioned according to the initial projection, and the predicted pose updates were used to reposition the C-arm to the predicted standard pose. During repositioning, first the detector translation was converted to a source translation, using the initial C-arm orientation and then, the angular parameters are inversely applied to the current C-arm pose (and inverted, respecting the laterality). Then, the predicted output can be simulated (Fig. 8, prediction). The second stage prediction cannot be evaluated retrospectively, because it lacks the real intermediate projection images. Figure 8 shows a visual comparison of the predicted standards and the associated reference standards, which were defined in the C-arm volume. For the *AP* standard (Fig. 8, top rows), the proposed method robustly predicts projections that are visually comparable to the reference standard. Also, for the *Lateral* standard (Fig. 8, bottom rows), the prediction results look promising and an iterative predicting procedure can potentially further refine the prediction result, as shown on the simulated dataset (Fig. 7). One example (Fig. 8, left) falls out due to low bone to tissue contrast, the femoral head is hardly visible. A comparison of the prediction results to the manually acquired standard projection illustrates an additional benefit of the proposed approach, which allows to automatically correct the in-plane rotation.

## Discussion

We have presented a deep learning-based sequential 2-stage framework for iteratively adjusting the C-arm pose to the desired standard projection. In contrast to the state of the art, the approach does not rely on any patient-individual prior information like preoperative CTs, instead we obtain the differential pose change for the C-arm directly from a first X-ray.

**Fig. 9:**
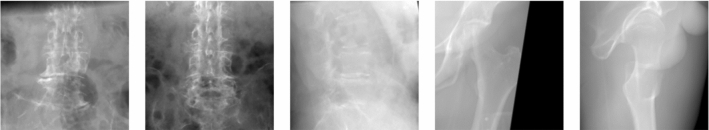
Standard projections of specific test patients that are related to the pose regression failure cases for different anatomies and standard projections

We demonstrated that an iterative 2-step approach for pose estimation significantly improves accuracy on synthetic images. The first stage regressor covers a broader capture range, which makes the approach more robust toward higher initial deviations, and complies the task of coarse alignment. The second stage fine-tunes the prediction and thereby improves accuracy. In clinical practice, this would reduce the continuous radiation to only two necessary acquisitions. Visually, predicted results are very similar to the reference X-ray images, which is most important for the surgeon. Furthermore, we analyzed the test images with a high pose variance after prediction and discovered that they mostly can be related to specific test patients. The corresponding reference standard projection is shown in Fig. 9 verifying that high pose errors are influenced by pathologies (arthrosis, obese) and artifacts (due to necessary volume cropping to prevent the projection of other extremities) not sufficiently represented in the training set. We think that further increasing the number of CT volumes in the training data has the potential to improve accuracy by representing more anatomical variations. Also random pixel or region dropout can improve the robustness of the pose regressor against projection artifacts.

Furthermore, we demonstrated that the regressor trained on *in silico* simulated DRRs also generalizes to unseen unfractured cadaver X-rays. The datasets of cadavers were available retrospectively; hence, the second stage prediction could not be evaluated on real X-rays because it lacks the real intermediate projection images. In the future, we plan to evaluate the complete 2-stage pipeline in a cadaver study. The high variance between accepted standard C-arm poses, observed in our trial on cadaver data, indicates that the qualitative measure does not allow for a direct assessment of the quality of the predicted standard projections. In addition, it confirms the usefulness of our method to help in standardizing the quality of good projections, especially in a highly dynamic clinical environment.

In our pose regression framework, we decided to model the C-arm orientation and the translation parallel to the C-arm detector plane and omit the translation along the C-arm beam direction and trained our pose regression model invariant toward image scaling. This is done for two reasons: (1) Translation along the beam direction is not relevant since it does not influence the projection plane. In clinical practice, the image intensifier is positioned as close to the patient as practical, which can be constrained due to patient anatomy or patient positioning. (2) The scale is influenced by patient size and can only be measured with respect to a reference when a preop CT is available. In our scenario, the availability of a 3D scan cannot always be assumed. In consequence, for our application, prediction of orientation and translation parallel to the detector plane is sufficient. The additional single degree of freedom can be altered freely while respecting constraints of the clinical environment and thus can facilitate handling anatomical restrictions in automatic C-arm positioning.

To translate the proposed method to the operating room will require addressing additional challenges related to pathologies, fractured bone and metal or instruments in the projections. For pathologies such as scoliosis, the method could be adapted to be trained and applied in a patch-based approach, where the field-of-view of the input patch is restricted to the region of one specific vertebra. Then, the method would only depend on the neighboring vertebra arrangements and could handle sideways curvature of the spine. In general, pose estimation on fractured bone is problematic because the regressor relies on the bone structure. But standard projections are used to monitor the implant/wire position. This means that bone fragments are already relocated to their initial position by inserting wires or implants. Thus, the fragment locations of the bone are very similar to healthy bone. In our future work, we will concentrate on the metal itself, that can partially overlay the anatomy. We plan to model this in our training data using metal simulation techniques [8, 14]. Moreover, path planning and handling obstacles in the operating room is an additional challenge that needs to be considered in the future work.

In conclusion, we have presented a deep learning-based sequential 2-stage framework for iteratively adjusting the C-arm pose to the desired standard projection directly from a first X-ray, without the need for prior CT scans to derive a pose model. The fully automatic approach generalizes well to different anatomies and standard projections and has the potential to seamlessly integrate into the clinical workflow, helping to standardize the quality of good projections, hence improving the quality of interventions while reducing time and dose.
