# Correction to: Toward automatic C‑arm positioning for standard projections in orthopedic surgery

**DOI:** 10.1007/s11548-021-02446-6

**Published:** 2021-07-18

**Authors:** Lisa Kausch, Sarina Thomas, Holger Kunze, Maxim Privalov, Sven Vetter, Jochen Franke, Andreas H. Mahnken, Lena Maier‑Hein, Klaus Maier‑Hein

**Affiliations:** 1 Division of Medical Image Computing, German Cancer Research Center, Heidelberg, Germany; 2 Division of Computer Assisted Medical Interventions, German Cancer Research Center, Heidelberg, Germany; 3 Imaging and Therapy Systems Division, Siemens Healthineers, Erlangen, Germany; 4 Medical Imaging and Navigation in Trauma and Orthopedic Suregery Research Group, BG Trauma Center, Ludwigshafen, Germany; 5 Division of Diagnostic and Interventional Radiology, Universitätsklinikum Marburg, Marburg, Germany

## Correction to: International Journal of Computer Assisted Radiology and Surgery (2020) 15:1095–1105 https://doi.org/10.1007/s11548-020-02204-0

The article Toward automatic C‑arm positioning for standard projections in orthopedic surgery, written by Lisa Kausch, Sarina Thomas, Holger Kunze, Maxim Privalov, Sven Vetter, Jochen Franke, Andreas H. Mahnken, Lena Maier‑Hein and Klaus Maier‑Hein, was originally published Online First without Open Access. After publication in volume 15, issue 7, page 1095–1105 the author decided to opt for Open Choice and to make the article an Open Access publication. Therefore, the copyright of the article has been changed to © The Author(s) 2021 and this article is licensed under a Creative Commons Attribution 4.0 International License, which permits use, sharing, adaptation, distribution and reproduction in any medium or format, as long as you give appropriate credit to the original author(s) and the source, provide a link to the Creative Commons licence, and indicate if changes were made. The images or other third party material in this article are included in the article’s Creative Commons licence, unless indicated otherwise in a credit line to the material. If material is not included in the article’s Creative Commons licence and your intended use is not permitted by statutory regulation or exceeds the permitted use, you will need to obtain permission directly from the copyright holder. To view a copy of this licence, visit http://creativecommons.org/licenses/by/4.0/.

The original article has been corrected.

Open Access funding enabled and organized by Projekt DEAL.

